# Effect of norepinephrine on the vascular waterfall and tissue perfusion in vasoplegic hypotensive patients: a prospective, observational, applied physiology study in cardiac surgery

**DOI:** 10.1186/s40635-023-00539-x

**Published:** 2023-08-21

**Authors:** Stefan Andrei, Stéphane Bar, Maxime Nguyen, Bélaid Bouhemad, Pierre-Grégoire Guinot

**Affiliations:** 1 Anaesthesiology and Critical Care Department, Dijon Bourgogne University Hospital, 2 Bd Maréchal de Lattre de Tassigny, 21000 Dijon, France; 2 Anaesthesiology and Critical Care Department, Carol Davila University of Medicine, Eroii Sanitari Bvd, no. 8, sector 5, Bucharest, Romania; 3 Anaesthesiology and Critical Care Department, Amiens University Hospital, Amiens, France; 4 University of Burgundy Franche Comté, LNC UMR1231, 21000 Dijon, France

**Keywords:** Vascular waterfall, Vasoplegia, Shock, Sepsis, Norepinephrine, ICU

## Abstract

**Background:**

Norepinephrine is a commonly used drug for treating vasoplegic acute circulatory failure in ICU. The prediction of norepinephrine macro- and micro-circulatory response is complicated by its uneven receptors’ distribution between the arterial and the venous structures, and by the presence of a physiological vascular waterfall (VW) that disconnects the arterial and the venous circulation in two pressure systems. The objectives of this study were to describe the VW in patients with arterial hypotension due to vasodilatory circulatory shock, and its behavior according to its response to norepinephrine infusion.

**Methods:**

A prospective, observational, bi-centric study has included adult patients, for whom the physician decided to initiate norepinephrine during the six first hours following admission to the ICU after cardiac surgery, and unresponsive to a fluid challenge. The mean systemic pressure (MSP) and the critical closing pressure (CCP) were measured at inclusion and after norepinephrine infusion.

**Results:**

Thirty patients were included. Norepinephrine increased arterial pressure and total peripheral resistances in all cohort. The cohort was dichotomized as VW responders (patients with a change of VW over the least significant change (≥ 93% increase in VW)), and as VW non-responders. In 19 (63%) of the 30 patients, VW increased from 3.47 [− 14.43;7.71] mmHg to 43.6 [25.8;48.1] mmHg, *p* < 0.001) with norepinephrine infusion, being classified as VW responders. The VW responders improved cardiac index (from 1.8 (0.6) L min^−1^ m^−2^ to 2.2 (0.5) L min^−1^ m^−2^, *p* = 0.002), capillary refill time (from to 4.2 (1.1) s to 3.1 (1) s, *p* = 0.006), and pCO_2_ gap (from 9 [7;10] mmHg to 6 [4;8] mmHg, *p* = 0.04). No baseline parameters were able to predict the VW response to norepinephrine. In comparison, VW non-responders did not significantly change the VW (from 5 [-5;16] mmHg to -2 [-12;15] mmHg, *p* = 0.17), cardiac index (from 1.6 (0.3) L min^−1^ m^−2^ to 1.8 (0.4) L min^−1^ m^−2^, *p* = 0.09) and capillary refill time (from 4.1 (1) s to 3.7 (1.4), *p* = 0.44).

**Conclusions:**

In post-cardiac surgery patients with vasoplegic arterial hypotension, the vascular waterfall is low. Norepinephrine did not systematically restore the vascular waterfall. Increase of the vascular waterfall was associated with an improvement of laboratory and clinical parameters of tissue perfusion.

## Introduction

Acute vasodilatory circulatory shock is one of the common hemodynamic syndromes in the intensive care unit (ICU), characterized by arterial hypotension caused by the alteration of vascular vasomotor tone, and a consequent alteration of tissue perfusion. The treatment consists in vasopressor infusion to restore blood pressure and tissue perfusion, the most used being norepinephrine (NE) [1]. NE has several vascular and cardiac effects: it increases vascular and venous resistance, cardiac preload and inotropy, and reduces vascular capacitance. Nevertheless, not all the patients show the same cardiovascular response to NE infusion: the blood pressure constantly increases, whereas cardiac output (CO) might not increase in some patients [2]. Because of this reason, restoring blood pressure with NE infusion may not always be associated with an improvement of tissue perfusion [3].

In the classic flow–pressure relationship description of the cardiovascular system, the tissue perfusion pressure depends on CO, mean arterial pressure (MAP), central venous pressure (CVP), and systemic vascular resistance. However, the arterial and the venous circulation are disconnected in two pressure systems by a vascular waterfall (VW) [4]. According to the VW concept, the tissue blood flow depends on the pressure difference between the critical closure pressure (CCP) at the arteriolar side and the mean systemic pressure (MSP) at the venous side. The VW describes the relationship between a pressure difference (CCP- MSP) and the blood flow of the concerned tissue or organ. When the VW gradient is altered, the tissue blood flow does not depend on the VW, but rather depends on the arterio-venous pressure difference. Several studies confirmed the existence of the VW phenomenon in organs like the lung, the heart, the liver, or the limb [5, 6]. Animal studies also demonstrated that the VW may be altered by vasoactive medications or diseases like sepsis, or hemorrhage [7–10]. Studies regarding the VW in patients with vasodilatory circulatory shock and its behavior with vasoactive drugs are sparse. One study has measured the VW in ICU, and it has described the effect of fluid expansion on the VW [4].

NE has vascular effects both on arterial and on venous side, with an increase of arterial and venous pressure [11]. Nevertheless, the prediction of NE macro- and micro-circulatory response is complicated by the uneven NE receptors distribution between the arterial and the venous vessels, and by the VW presence, making the evaluation of vascular tone difficult [12]. The presence of VW might explain why in some cases, NE infusion may not improve tissue perfusion despite an increase of blood pressure. To date, we do not know how the VW is affected in patients with vasoplegic syndrome and/or by NE.

The main objective of this study was to describe the VW in patients with vasodilatory circulatory shock and its response to norepinephrine. The secondary objectives were to describe the hemodynamic differences between patients with significant changes of VW and these effects on indirect parameters of tissue perfusion.

## Material and methods

### Patients

A prospective observational study was performed in two University Hospital cardiovascular ICU. The study was approved by the national independent ethics committee (17/2017). All subjects received written information about the study and provided their consent to participate prior to cardiac surgery. The study complied with the tenets of the Declaration of Helsinki.

Inclusion criteria were: adult patients (≥ 18 years), monitoring with a central venous access and invasive blood pressure, for whom the physician decided to initiate a norepinephrine infusion during the six first hours following admission to the ICU after cardiac surgery, and unresponsiveness of blood pressure to a fluid challenge [13]. Non-inclusion criteria were cardiac arrhythmia, cardiac conduction disorders, permanent or temporary pacemaker, prior (intra- or post-operative) inotropic or vasopressor drug infusion, right heart dysfunction (defined by a right ventricular fractional area change (RVFAC) lower than 35%), the presence of extracorporeal membrane oxygenation (ECMO), and the presence of left ventricle or right ventricle assistance devices (LVAD or RVAD).

### Hemodynamic parameters

The systolic blood pressure (SAP), diastolic blood pressure (DAP) and mean blood pressure (MAP) were measured using an invasive arterial catheter. Transthoracic echocardiography (CX50 Ultrasound System and an S5-1 Sector Array Transducer, Philips Medical System, Suresnes, France) was performed by a board-certified physician. Left ventricular ejection fraction (LVEF) was calculated using Simpson’s method on a four-chamber view. The diameter of the left ventricular outflow tract was measured on a long-axis parasternal view at the time of patient inclusion. The aortic velocity–time integral (VTIAo) was measured with pulsed Doppler on a five-chamber apical view. Stroke volume (SV; mL) was calculated as VTIAo × aortic area and was expressed as indexed SV (SVi) = SV/body surface area (ml.m^−2^). Cardiac index (CI) (l.min^−1^.m^−2^) was calculated as SVi × heart rate (HR). Mean echocardiographic parameters were calculated from the average over five consecutives cardiac cycles and analyzed retrospectively.

The mean systemic pressure (MSP) and CCP were measured using the inspiratory breath-hold maneuver, as previously described [12], within the first 2 h after postoperative ICU admission. The ventilatory mode was switched to pressure assist control, allowing for inspiratory hold maneuvers. Steady-state arterial pressure, CVP and cardiac output were measured considering the last 3 s of 12-s inspiratory hold maneuvers using plateau pressures of 5, 15, 25 and 35 cmH_2_O. After the inspiratory hold initiation, a steady state was noted between 7 and 12 s. All reported values were the average of the values recorded within the last 3 s. For the four inspiratory hold procedures, measured venous and arterial pressures were plotted against cardiac output, and a linear regression line was fitted to the data points. The MSP was determined by extrapolation of the CVP to zero flow on the venous return curve. The CCP was determined by extrapolation of the arterial pressure to zero flow on the ventricular output curve. The VW was calculated as the difference CCP-MSP.

Indexed systemic arterial resistance (SARi, mmHg ml^−1.^m^−2^) was calculated as (MAP—CCP)/CI. The indexed total peripheral resistance (TPRi) was calculated as (MAP-CVP)/CI (mmHg ml^−1.^m^−2^).

Arterial–venous CO_2_ partial pressure difference (pCO_2_ gap) and arterial–venous oxygen tension difference (CavO_2_) were calculated on arterial and venous blood gases as follows: CaO_2_ = 1.34 × Hb x SaO_2_ + 0.003 × PaO_2_; CvO_2_ = 1.34 × Hb x ScvO_2_ + 0.003 × PvO_2_, where Hb is the hemoglobin concentration (g dl^−1^), PaO_2_ is the arterial oxygen pressure (mmHg), SaO_2_ is the arterial oxygen saturation (%), PvO_2_ is the venous oxygen pressure (mmHg), ScvO_2_ is the central venous oxygen saturation (%), and 0.003 is the solubility coefficient of oxygen, pCO_2_ gap = PcvCO_2_–PaCO_2_ (mmHg), C(a-v)O_2_ = CaO_2_-CvO_2_ (ml) [14].

### Study protocol

At the inclusion, clinical and demographical parameters were collected: age, gender, weight, height, comorbidities, clinical scores, ventilation parameters, type of surgery. Measurements of capillary refill time (sec), HR, CVP, SAP, MAP, DAP, CVP, TPRi, SVi, CI, and vascular determinants of waterfall were noted, and arterial/venous blood gas analyses were performed at the baseline, before norepinephrine initiation. Norepinephrine used in this study was norepinephrine tartrate. The protocol of norepinephrine infusion was standardized as follows: a syringe (50 ml) of norepinephrine (dilution of 0.1 mg ml^−1^) was continuously infused with a starting dose of 0.5 ml h^−1^. The posology (step of 0.1 ml h^−1^) was adapted to obtain a MAP over 65 mmHg. All the hemodynamic measurements were performed again after 15 min of hemodynamic stability defined as a variation of MAP less than 10% with norepinephrine infusion. Capillary refill time (s) was measured at the distal phalanx of the index finger, and comprises the average of three measures [15].

The ventilatory parameters and sedation conditions remained unchanged between the two rounds of measurements. All patients were sedated by continuous infusion of propofol, fully adapted to ventilator with one intravenous bolus of cisatracurium (0.15 mg.kg^−1^), and under controlled mechanical ventilation with a tidal volume (Vt) set at 7–9 ml.kg^−1^ of ideal body weight, and a positive end-expiratory pressure (PEEP) of 5–8 cmH_2_O. No spontaneous breathing efforts were observed during the measurements.

### Statistical analyses

Normality was visually assessed using histograms and QQ plots. Accordingly, quantitative data are presented as medians (interquartile range), mean (standard deviation), and qualitative data are presented as frequencies (percentages). To define VW response, we calculated the coefficient of variation and the least significant change of the VW. The least significant change is the minimal change observed that can be considered as real and not related to the variability of the measurement. In other words, the least significant change is the minimal change that needs to be measured to recognize a real change of the variable. This measure includes the coefficient error (coefficient error = coefficient of variation/√2), and it is described as follows: least significant change (%) = coefficient error × 1.96 × √2. The coefficient of variation (66%) and the least significant change (93%) of the VW were calculated on baseline values, as suggested by other authors [16]. In the absence of any clear previous data, we planned to include 30 patients. Based on the least significant change of the VW and a mean VW value of 35 (17) mmHg, a sample size of 19 patients can demonstrate a significant change of the VW with a power of 90% and a risk alpha of 0.05. The population was dichotomized as VW responders, i.e., patients with a change of VW over the least significant change (≥ 93% increase in VW), and VW non-responders. Non-parametric or parametric tests were performed for mean comparisons and for correlation evaluation, as appropriate. A matrix of correlation using bivariate Pearson’s correlation tests between the different baseline hemodynamic parameters and the hemodynamic parameters change with norepinephrine infusion was constructed. Statistical analyses were performed using R software version 3.4.4 (R Foundation for Statistical Computing, Wien, Austria). The threshold for statistical significance was set to *p* < 0.05.

## Results

Of the 55 patients screened during the study period, 30 patients were included and analyzed in the study. The study population characteristic is summarized in Table 1. The mean age was 69 (9) years, with 77% males, the most prevalent comorbidities being arterial hypertension, dyslipidemia, and valvular heart disease. Most patients underwent valvular and coronary artery bypass graft surgery. The hemodynamic characteristics at the baseline and after the initiation of NE infusion are shown in Table 2. Baseline body temperature was 36.5 (0.5) °C.

**Table 1:** General characteristics for the study population

Characteristics	All patients (n = 30)
Age (years), mean (SD)	68.5 (9.23)
Sex (male), *n* (%)	23 (76.7%)
Weight (kg), mean (SD)	87.3 (19.3)
SAPS II, mean (SD)	41.8 (11.8)
NE infusion rate (mcg kg^−1^ min^−1^), median [IQR]	0.09 [0.07; 0.12]
Cumulative fluid infusion before NE infusion (ml kg^−1^), mean (SD)	9.30 (3.51)
Comorbidities	
High blood pressure, *n* (%)	23 (76.7%)
Diabetes, *n* (%)	9 (30.0%)
Dyslipidemia, *n* (%)	13 (43.3%)
Chronic kidney disease, *n* (%)	2 (6.67%)
COPD, *n* (%)	3 (10.0%)
Smoking history, *n* (%)	6 (20.0%)
Respiratory status	
Tidal volume (ml/kg), median [IQR]	8 [7; 8]
PEEP (cmH20), median [IQR]	6 [5; 8]
Type of surgery, *n* (%)	
- CABG	5 (16.7%)
- Valve surgery	16 (53.3%)
- Mixed	4 (13.3%)
- Other	5 (16.7%)

*CABG* coronary artery bypass graft, *COPD* chronic obstructive pulmonary disease, *IQR* 25%-75% interquartile range, *NE *norepinephrine, *PEEP* positive end-expiratory pressure, *SAPS II* Simplified Acute Physiology Score (SAPS) II, *SD* standard deviation

**Table 2:** Comparison of hemodynamic parameters before and after intervention for VW-responders and non-responders patients (^1^Wilcoxon matched-pair signed rank test, *p*-value shown)

Variable	Baseline	After NE	p-value^1^
Heart rate (bpm), mean (SD)			
All cohort	81 (18)	81 (18)	0.791
- VW responders	82 (20)	82 (20)	0.72
- VW non-responders	79 (13)	79 (15)	0.87
Systolic arterial pressure (mmHg), mean (SD)			
All cohort	85 (10)	121 (15)	< 0.001
- VW responders	86 (9)	124 (17)	< 0.001
- VW non-responders	84 (12)	116 (8)	< 0.001
Mean arterial pressure (mmHg), mean (SD)			
All cohort	59 (7)	80 (12)	< 0.001
- VW responders	60 (5)	85 (13)	< 0.001
- VW non-responders	58 (9)	77 (8)	< 0.001
Diastolic arterial pressure (mmHg), mean (SD)			
All cohort	47 (7)	63 (12)	< 0.001
- VW responders	47 (6)	65 (13)	< 0.001
- VW non-responders	46 (9)	58 (9)	< 0.001
Central venous pressure (mmHg), mean (SD)			
All cohort	7 (4)	9 (4)	< 0.001
- VW responders	7 (4)	9 (4)	< 0.001
- VW non-responders	8 (4)	10 (4)	0.01
Mean systemic pressure (mmHg), median [IQR]			
All cohort	23 [18; 26]	22 [15; 26]	0.269
- VW responders	22 [17; 26]	20 [16; 25]	0.15
- VW non-responders	23 [17; 26]	24 [22; 28]	0.59
Critical closure pressure (mmHg), median [IQR]			
All cohort	25 [14; 34]	45 [27;60]	< 0.001
- VW responders	26 [20; 29]	58 [46;71]*	< 0.001
- VW non-responders	25 [18 ;37]	27 [15;40]*	0.58
Vascular waterfall (mmHg), median [IQR]			
All cohort	3.5 [**− **10; 10]	23 [− 2; 47]	< 0.001
- VW responders	4 [**− **14; 8]	44 [26; 48]*	0.001
- VW non-responders	5 [**− **5; 16]	− 2 [− 12; 15]*	0.17
Indexed systemic arterial resistance (mmHg L^−1^ min^−1^ m^−2^), mean (SD)			
All cohort	20 [14; 28]	16 [7; 29]	0.271
- VW responders	19 [14; 34]	11 [5; 24]*	0.002
- VW non-responders	22 [6; 26]	28 [16; 32]*	0.03
Indexed total peripheral resistances (mmHg L^−1^ min^−1^ m^−2^), mean (SD)			
All cohort	34 (17)	40 (15)	< 0.001
- VW responders	34 (21)	40 (18)	0.002
- VW non-responders	34 (9)	40 (8)	0.013
Cardiac index (L min^−1^ m^−2^), mean (SD)			
All cohort	1.7 (0.6)	2 (0.5)	< 0.001
- VW responders	1.8 (0.6)	2.2 (0.5)*	0.002
- VW non-responders	1.6 (0.3)	1.8 (0.4)*	0.09
ScvO_2_ (%), mean (SD)			
All cohort	61 (10)	67 (10)	0.001
- VW responders	64 (10)	69 (8)	0.02
- VW non-responders	57 (8)	63 (12)	0.05
pCO_2_ gap (mmHg), median [IQR]			
All cohort	9 [7; 11]	7 [4; 10]	0.073
- VW responders	9 [7; 10]	6 [4; 8]*	0.04
- VW non-responders	10 [8.00; 11]	11 [8; 12]*	0.57
pCO_2_ gap/arterio-venous oxygen tension, median [IQR]			
All cohort	1.8 [1.6; 2.1]	1.9 [0.9; 2.3]	0.289
- VW responders	1.9 [1.6; 2.1]	1.6 [0.8; 2.1]	0.04
- VW non-responders	1.8 [1.6; 2.0]	2.2 [1.8; 2.5]	0.38
Arterial lactates (mmol L^−1^), mean (SD)			
All cohort	1.6 (0.5)	1.7 (0.7)	0.705
- VW responders	1.5 (0.4)	1.6 (0.6)	0.52
- VW non-responders	1.8 (0.6)	1.8 (0.7)	0.71
Capillary refill time (s), mean (SD)			
All cohort	4.2 (1.1)	3.3 (1.1)	0.001
- VW responders	4.2 (1.1)	3.1 (1)	0.006
- VW non-responders	4.1 (1)	3.7 (1.4)	0.44

At each timepoint the two groups were also compared (Wilcoxon signed rank test, p-value not shown; * = statistically significant difference between the VW-responders and non-responders patients)

*bpm* beats per minute, *IQR* 25%-75% interquartile range, *NE* norepinephrine, *pCO*_*2*_ carbon dioxide pressure, *ScvO*_*2*_ central venous oxygen saturation, *SD* standard deviation, *VW* vascular waterfall

### Whole cohort

At baseline the CCP was 25 [14; 34] mmHg, the MSP was 22 [17; 26] mmHg, the CVP was 7 (4) mmHg. The resulting VW was 4 [-10;10] mmHg. The mean amount of fluid bolus did not differ between the two groups (9.3 (3.7) ml kg^−1^ vs 9.6 (3.2) ml kg^−1^, *p* = 0.755).

The NE infusion increased the mean arterial pressure from 59 (7) mmHg to 80 (12) mmHg (*p* < 0.001, Wilcoxon matched-pair signed rank test), the CVP from 7 (4) to 9 (4) mmHg (*p* < 0.001, Wilcoxon matched-pair signed rank test), the CI from 1.7 (0.6) L min^−1^ m^−2^ to 2 (0.5) L min^−1^ m^−2^ (*p* < 0.001, Wilcoxon matched-pair signed rank test), the indexed total peripheral resistances from 34 (17) mmHg L^−1^ min^−1^ m^−2^ to 40 (15) mmHg L^−1^ min^−1^ m^−2^ (*p* < 0.001, Wilcoxon matched-pair signed rank test), the CCP from 25 [14; 34] mmHg to 45 [27; 60] mmHg (*p* < 0.001, Wilcoxon matched-pair signed rank test) and the VW from 3.5 [− 10;10] mmHg to 23 [− 2;47] mmHg (*p* < 0.001, Wilcoxon matched-pair signed rank test). Other parameters like the MSP did not statistically significantly change from 23 [18; 26] mmHg to 22 [15; 26] mmHg (*p* = 0.269, Wilcoxon matched-pair signed rank test).

### Vascular waterfall responders

**Fig. 1:**
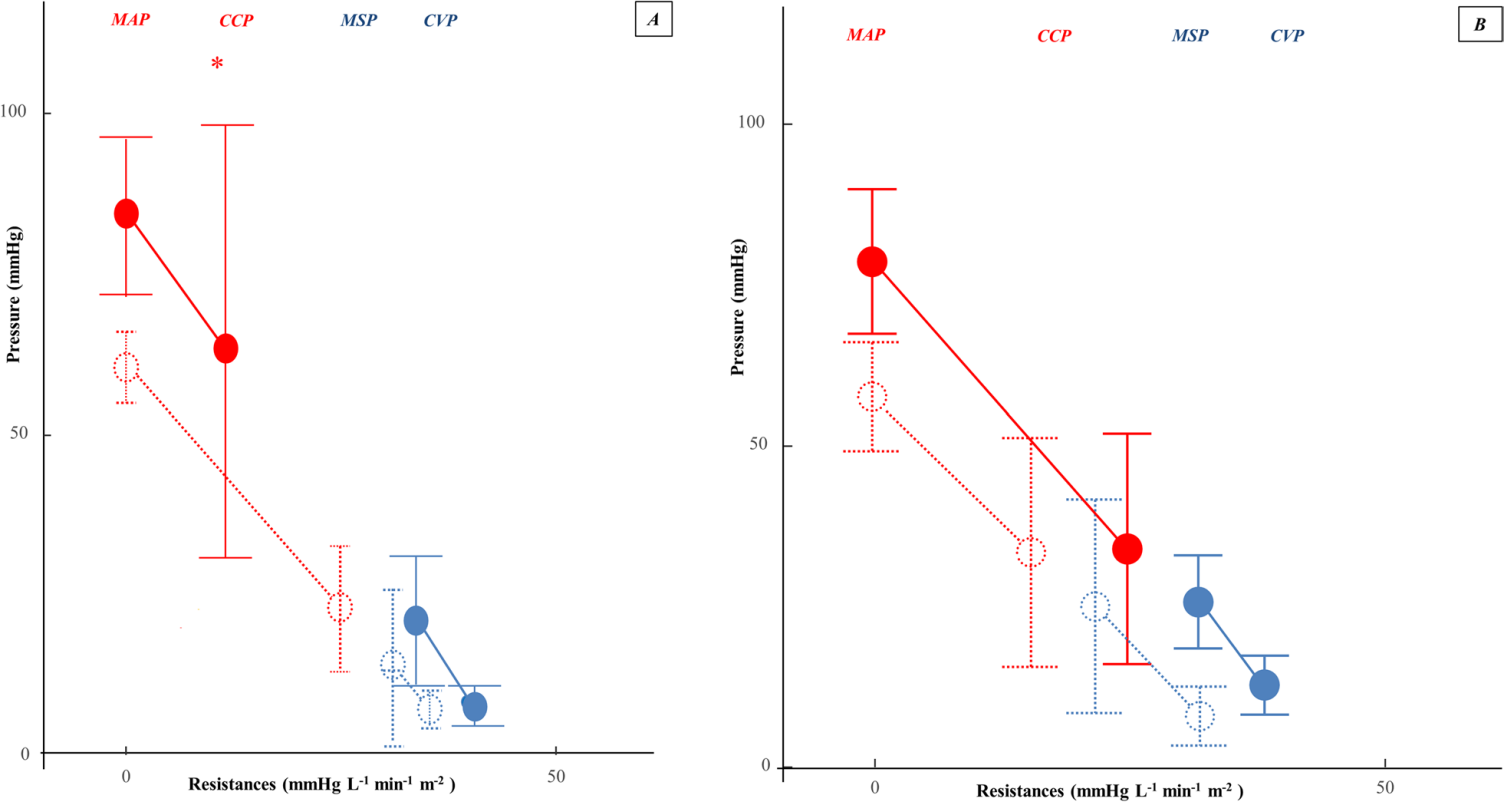
Norepinephrine effects on MAP (mean arterial pressure), CCP (critical closing pressure), MSP (mean systemic pressure), and CVP (central venous pressure), presented comparatively for VW-responders (**A)** and VW-non-responders (**B**). Norepinephrine significantly restores the VW (CCP – MSP) only in VW-responders. Statistical comparison of median values use the Wilcoxon matched-pair signed rank test. *Statistically significant increase in CCP comparing with baseline

In 19 (63%) of the 30 patients, VW increased from 3.47 [− 14.43; 7.71] mmHg to 43.6 [25.8;48.1] mmHg, *p* < 0.001, Wilcoxon matched-pair signed rank test) with NE. These patients were classified as VW-responders (Fig. 1). CCP increased from 25.7 [9.67; 29.2] mmHg to 57.6 [45.5; 70.9] mmHg with NE in VW-responders (p < 0.001, Wilcoxon matched-pair signed rank test). NE increased blood pressure (MAP from 60 (5) mmHg to 85 (13) mmHg, *p* < 0.001, Wilcoxon matched-pair signed rank test), CVP (from 7 (4) mmHg to 9 (4), p = 0.01, Wilcoxon matched-pair signed rank test), CI (from 1.8 (0.6) L min^−1^ m^−2^ to 2.2 (0.5) L min^−1^ m^−2^, p = 0.002, Wilcoxon matched-pair signed rank test), TPRi (from 34 (21) mmHg min^−1^ m^−2^ to 40 (18) mmHg min^−1^ m^−2^, p = 0.002, Wilcoxon matched-pair signed rank test), SvO_2_ (from 64 (10) % to 69 (8) %, *p* = 0.02, Wilcoxon matched-pair signed rank test), and decreased SRAi (from 19 [14;34] mmHg min^−1^ m^−2^ to 11 [5;24] mmHg min^−1^ m^−2^, *p* = 0.002, Wilcoxon matched-pair signed rank test), capillary refill time (from to 4.2 (1.1) s to 3.1 (1) s, p = 0.006), pCO_2_ gap (from 9 [7; 10] mmHg to 6 [4; 8] mmHg, *p* = 0.04, Wilcoxon matched-pair signed rank test) and pCO_2_ gap/arterio-venous oxygen tension (from 1.9 [1.6; 2.1] to 1.6 [0.8; 2.1], *p* = 0.04, Wilcoxon matched-pair signed rank test).

### Vascular waterfall non-responders

NE increased blood pressure (MAP from 58 (9) mmHg to 77 (8) mmHg, *p* < 0.001, Wilcoxon matched-pair signed rank test), TPRi (from 34 (9) mmHg min^−1^ m^−2^ to 40 (8) mmHg min^−1^ m^−2^, p = 0.013, Wilcoxon matched-pair signed rank test) and SRAi (from 22 [6; 26] mmHg min^−1^ m^−2^ to 28 [16; 32] mmHg min^−1^ m^−2^, *p* = 0.03, Wilcoxon matched-pair signed rank test). VW (from 5 [− 5; 16] mmHg to -2 [− 12; 15] mmHg, *p* = 0.17, Wilcoxon matched-pair signed rank test), CCP (from 25 [18;37] mmHg to 27 [15;40] mmHg, *p* = 0.58, Wilcoxon matched-pair signed rank test), CI (from 1.6 (0.3) L min^−1^ m^−2^ to 1.8 (0.4) L min^−1^ m^−2^, *p* = 0.09, Wilcoxon matched-pair signed rank test), tissue perfusion parameters (Table 2), and capillary refill time (from 4.1 (1) s to 3.7 (1.4), *p* = 0.44, Wilcoxon matched-pair signed rank test) did not significantly change.

### Prediction of vascular waterfall changes

**Fig. 2:**
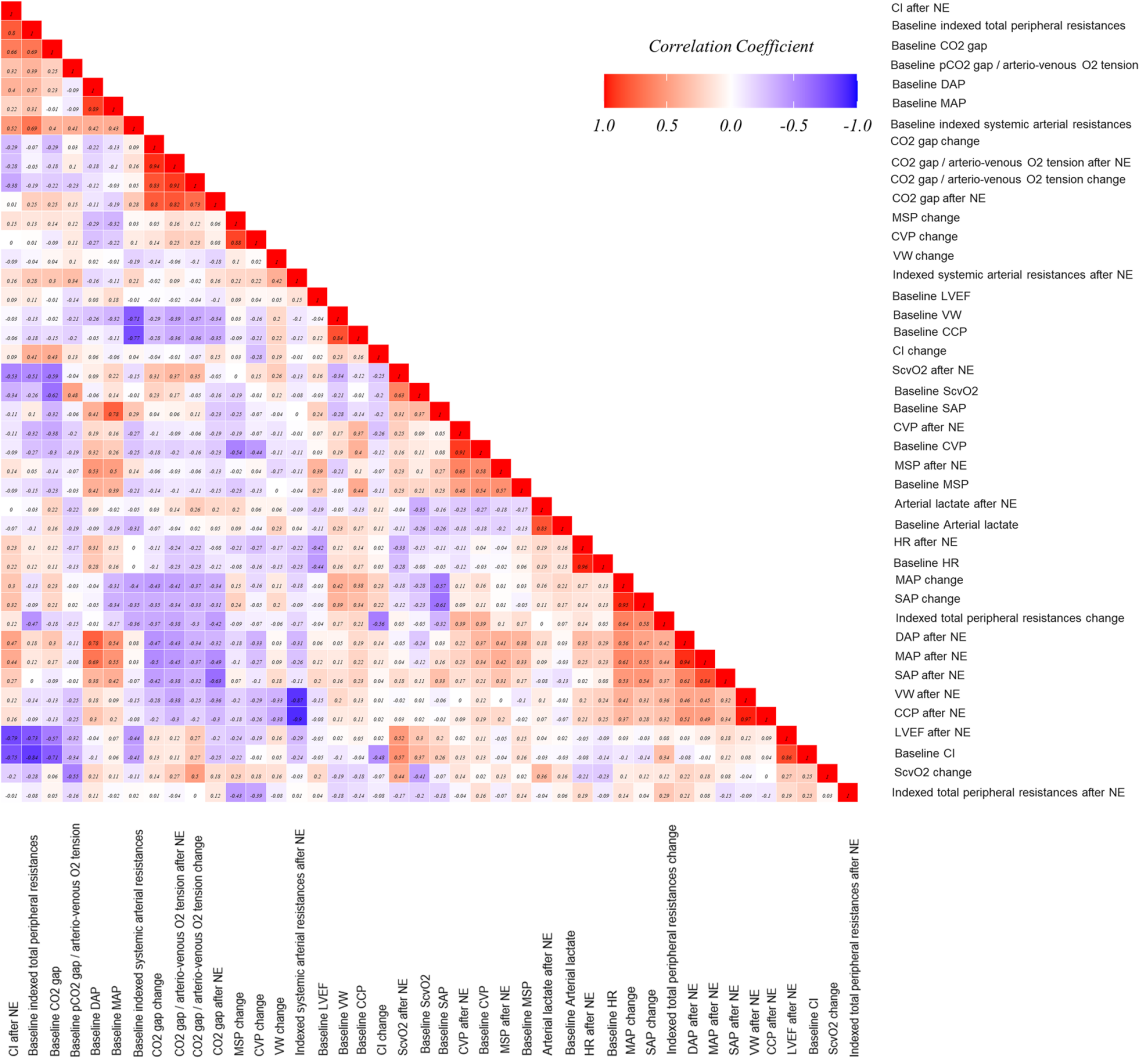
Visual representation of the matrix of correlation using bivariate Pearson’s correlation tests between the different baseline hemodynamic parameters and the hemodynamic parameters change with norepinephrine infusion. The analyzed variables are those provided in Table 2. Suggestion of interpretation: we can notice the weak correlation (lack of color), thus, the unpredictable response based on baseline parameters. *CCP* critical closing pressure, *CI* cardiac index, *CVP* central venous pressure, *DAP* diastolic arterial pressure, *HR* heart rate, *LVEF* left ventricle ejection fraction, *MAP* mean arterial pressure, *SAP* systolic arterial pressure, *MSP* mean systemic filling pressure, *NE* norepinephrine, *VW* vascular waterfall

NE dose did not differ between the responders and non-responders' groups (0.08 [0.06; 0.12] gamma kg^−1^ min^−1^ vs 0.09 [0.06;1.1] gamma kg^−1^ min^−1^, *p* = 0.93, Wilcoxon signed rank test). At baseline, none of the hemodynamic (blood pressure, CVP, CI), and tissue perfusion parameters differed between the two groups (Table 2). Thus, none of the hemodynamic and tissue perfusion parameters were associated with the VW and its changes (*p* > 0.05). A graphic representation of an exploratory correlation matrix regarding the measured baseline hemodynamic parameters and their changes with NE infusion is provided in Fig. 2.

## Discussion

Patients with arterial hypotension owing to vasoplegia have inactive VW (the value is around 0). Despite systematically increasing blood pressure, NE only restored the VW in two-thirds of patients. Improvement of CI and tissue perfusion parameters was only observed in patients who have restored the VW. None of the hemodynamic parameters predicted the behavior of VW with NE infusion.

Our results add more knowledge on arterial hypotension, vasoplegia, VW and their response to NE. The VW was suggested to have a role in maintaining tissue (and organ) perfusion (17, 18, 19). We confirmed that the VW is altered during arterial hypotension owing to vasoplegia, as it was observed during experimental vasodilation [5]. The VW may be understood as a phenomenon that describes a relationship between pressure gradient and blood flow at a level of interest (organ, tissue, arterial side, venous side). The level of blood pressure at arteriolar and/or venous side may affect the behavior of the VW (the VW can be active or inactive) [20]. We observed a higher MSP value compared to CCP in some patients, resulting in negative VW. This finding is consistent with previous studies [9, 10], suggesting that blood flow might have an independent relationship with the VW. This observation appears counterintuitive, but it has already been observed in sepsis with this method [9], and it has been demonstrated in experimental studies [5, 20]. The VW measured in the present study may be understood as the sum of the VW of each tissue and organs of the body. However, it is important to note that different tissues and organs exhibit distinct VW values [6, 18], especially in the context of vasoplegia [5].

Shrier et al. [5] demonstrated that during maximal vasodilation (a situation similar to this observed in our study), the CCP can be less than the downstream pressure; the VW becoming very low or negative. In this case, the VW is abolished and the CCP does not affect the tissue blood flow, thus the tissue flow becomes dependent of the pressure gradient between the arterial and venous sides (i.e., MAP-CVP) [5]. These observations explain the contradictory literature on the perfusion pressure (i.e., MAP-CVP), the microcirculation, and organs’ function. Some studies have demonstrated an association between the perfusion pressure and the microcirculation, or the kidney function in septic patients [21]. Sepsis shock is characterized by vasodilation with an alteration of the VW: the blood flow becomes dependent of the perfusion pressure [9]. On the contrary, during anesthesia no correlation was demonstrated between the perfusion pressure and the microcirculation [22]. Patients are healthy and they do not have alteration of the VW. Our observations are in line with these points. Improvement of tissue perfusion parameters was only observed in patients who have increased the VW. Increase of VW was associated with a decrease of systemic arterial resistance and an increase of blood flow. These findings confirm the importance of the VW to maintain tissue perfusion.

One question is why some patients did not increase the VW. The main difference between responder and non-responder patients was the CCP that has only increased in responder patients. In non-responder patients, we observed an increase of arteriolar tone with NE infusion, suggesting that increases of blood flow and blood pressure were unbalanced. Because the increase of blood flow was too low to counterbalance the NE induced changes of vascular properties (vessel compliance, vascular tone), the arteriolar tone has more increased than blood flow. In such situation, tissue perfusion could be compromised as we observed sustained abnormal values of pCO_2_ gap that reflect persistent abnormal tissue blood flow [14]. In the VW non-responder group, the CI was lower than the threshold defining low cardiac output syndrome (and slightly lower than in VW responder group, although not statistically different). Due to the observational study design, we cannot definitively conclude on the mechanisms explaining why NE did not increase the VW in some patients, and on the causal relationship between the VW, the CI and the parameters of tissue parameters.

Our results bring further explanation regarding the underlying physiological mechanism related to the effects of NE on the VW, pressure tissue perfusion and blood flow tissue. The main effect seemed to be taken by the arterial determinant of VW, as the MSP was not modified by the NE infusion. The effect of NE on the venous side is a dose dependent phenomenon. The MSP did not change because the NE dose was lower than the dose associated with changes of the venous return and preload [23]. The end-arteriolar pressure (i.e., CCP) was decreased in case of blood flow and blood pressure increase. This finding is different from that observed with infusion of esmolol in late phase of septic shock patients [9]. But our results are complementary to previous results. Taken altogether, these results suggest that VW may be manipulated in different way according to the type and the phase of shock, and the type of vasoactive medication administrated. These findings are in accordance with the concept of coherence between the macro-circulation and the micro-circulation [24]. Studies have demonstrated that arteriolar tone depends of several systemic (neuro-vegetative, hypoxia), local, endothelial and flow mediated factors [24–29]. Moreover, the VW can be affected by external factors such as interstitial fluid pressure and extra mural forces of the vessel that are altered in ICU patients. Because the underlying disease and the treatment can alter these factors in different ways, because each organ also may be affected in different way, and these effects are not predictable, the effects of hemodynamic treatment on the VW and tissue perfusion are not predictable.

### Clinical relevance and perspectives

Our findings strengthen the importance of monitoring the coherence between (and within parameters of) macro-circulation (blood pressure, blood flow) and micro-circulation when physician use vasoactive treatment in order to improve tissue perfusion. NE may be associated with alteration of tissue blood flow despite an increase in blood pressure because of unbalance between flow and pressure at the level of the tissue [30]. This result is in accordance with a previous study demonstrating that dynamic arterial elastance (which is the ratio between dynamic change of pulse pressure and blood flow) was associated with the VW [12]. The higher was the dynamic arterial elastance, the lower was the VW, suggesting that when pressure changes are higher than those of blood flow, the VW may be compromised. These observations may explain the findings of studies demonstrating an association between NE exposure and organ dysfunction [30, 31], or the studies that failed to demonstrate better clinical outcomes with higher target of blood pressure [32]. Considering the difficulties of VW assessment at bedside, physician should use indirect methods that were demonstrated to be associated with the VW, the tissue blood flow and the microcirculation, such as the measure of dynamic arterial elastance, ventriculo-arterial coupling, and/or indirect parameters of tissue perfusion (capillary refill time, pCO_2_ gap) [2], [34, 35]. The ratio PvaCO_2_/CavO_2_ decreases in VW responders, suggesting that this group might have a reduction in tissue hypoxia after NE infusion. To simplify the message, the persistence of high arterial lactate, pCO_2_ gap value or capillary refill time values despite normalization of blood pressure with NE infusion, should lead to evaluate cardiac output and/or to reconsider the dose of NE. Blood flow may be too low with regard to the blood pressure. A further step might be to add an inodilator drug to increase blood flow when tissue perfusion parameters are not completely restored with NE (i.e., in VW non-responder group).

Because we have evaluated the VW as a whole and we did not measure other possible explicative parameters [36] (such as sublingual microcirculation or organ function), we cannot totally conclude on this issue. It remains to be demonstrated how monitoring VW and global markers of tissue perfusion can help to optimize vasopressor use and their effects on tissue perfusion.

### Limitations

The present study is limited by the low number of patients. The study could be underpowered to detect some correlations and differences, whereas the sample size is enough to assess VW changes. Firstly, one question concerns the method used to measure the VW and CCP. This method was previously demonstrated to be reasonably accurate, reliable, and reproducible [4, 9, 11, 12]. Secondly, since the VW is calculated by a method using MAP, CVP and CI we cannot rule out the potential of mathematical coupling between these variables, the VW, and the indexed systemic arterial resistance. As already mentioned, this technique was previously used at bedside [4, 9, 11], and the observed VW and CCP values are closed to those previously measured [4, 9]. The aim of this study was to describe the VW in the context of vasoplegia and NE use, as it was previously performed for venous return curves [11]. We did not measure variables of organ function or injury (such as serum creatinine, troponin) to demonstrate a link between VW alterations and organ function. Finally, we analyzed a cohort within the specific context of postoperative cardiac surgery, and we used a clinical pragmatic definition of arterial hypotension owing to vasoplegia. We cannot totally exclude arterial hypotension in relation to alteration of inotropy. Consequently, the results may be further validated in other types of shock.

## Conclusion

In post-cardiac surgery patients with vasoplegic hypotension requiring low-dose vasopressor, the VW is low. Norepinephrine restores the VW in only two-thirds of patients. The increase of the VW was associated with an increase of tissue perfusion parameters.

## Data Availability

The collected dataset for this study can be made available by the corresponding author upon reasonable request.

## Abbreviations

CCP: Critical closing pressure
CI: Cardiac index
CO: Cardiac output
CVP: Central venous pressure
DAP: Diastolic arterial pressure
ECMO: Extracorporeal membrane oxygenation
HR: Heart rate
ICU: Intensive care unit
LVAD: Left ventricle assistance device
LVEF: Left ventricular ejection fraction
MAP: Mean arterial pressure
MSP: Mean systemic pressure
NE: Norepinephrine
PP: Pulse pressure
TPRi: Indexed total peripheral resistance
RVAD: Right ventricle assistance device
RVFAC: Right ventricular fractional area change
SAP: Systolic arterial pressure;
SARi: Indexed systemic arterial resistance
SV: Stroke volume
SVi: Indexed stroke volume;
VRi: Indexed systemic venous resistance
VTIAo: Aortic velocity–time integral
WF: Vascular waterfall
